# Protocol: High-throughput and quantitative assays of auxin and auxin precursors from minute tissue samples

**DOI:** 10.1186/1746-4811-8-31

**Published:** 2012-08-10

**Authors:** Xing Liu, Adrian D Hegeman, Gary Gardner, Jerry D Cohen

**Affiliations:** 1 Plant Biological Sciences Graduate Program, Department of Horticultural Science, and Microbial and Plant Genomics Institute, University of Minnesota, 1970 Folwell Avenue, Saint Paul, MN, 55108, USA; 2 Division of Biology, 156–29, California Institute of Technology, Pasadena, CA, 91125, USA

## Abstract

**Background:**

The plant hormone auxin, indole-3-acetic acid (IAA), plays important roles in plant growth and development. The signaling response to IAA is largely dependent on the local concentration of IAA, and this concentration is regulated by multiple mechanisms in plants. Therefore, the precise quantification of local IAA concentration provides insights into the regulation of IAA and its biological roles. Meanwhile, pathways and genes involved in IAA biosynthesis are not fully understood, so it is necessary to analyze the production of IAA at the metabolite level for unbiased studies of IAA biosynthesis.

**Results:**

We have developed high-throughput methods to quantify plant endogenous IAA and its biosynthetic precursors including indole, tryptophan, indole-3-pyruvic acid (IPyA), and indole-3-butyric acid (IBA). The protocol starts with homogenizing plant tissues with stable-labeled internal standards added, followed by analyte purification using solid phase extraction (SPE) tips and analyte derivatization. The derivatized analytes are finally analyzed by selected reaction monitoring on a gas chromatograph-mass spectrometer (GC-MS/MS) to determine the precise abundance of analytes. The amount of plant tissue required for the assay is small (typically 2–10 mg fresh weight), and the use of SPE tips is simple and convenient, which allows preparation of large sets of samples within reasonable time periods.

**Conclusions:**

The SPE tips and GC-MS/MS based method enables high-throughput and accurate quantification of IAA and its biosynthetic precursors from minute plant tissue samples. The protocol can be used for measurement of these endogenous compounds using isotope dilution, and it can also be applied to analyze IAA biosynthesis and biosynthetic pathways using stable isotope labeling. The method will potentially advance knowledge of the role and regulation of IAA.

## Introduction

Auxin, the first discovered plant hormone, plays critical roles in plant growth, organ formation, and plant responses to environmental stimuli. As the major form of natural auxin, indole-3-acetic acid (IAA) has been extensively studied, and mechanisms of its function and regulation are being revealed. To trigger downstream signaling responses, IAA functions like molecular glue, which ties its receptor TIR1, an F-box protein, with Aux/IAA transcriptional repressors, leading to degradation of Aux/IAA and thus releasing the transcriptional suppression of auxin responsive genes [1]. Based on this functional mechanism, auxin responses in cells can be partially controlled by the cellular concentration of IAA. Because IAA is a mobile signaling molecule that can be transported among cells to form auxin gradients and auxin maxima that are essential for plant development [2], a method that allows quick and precise measurement of IAA in specific plant tissues will greatly facilitate understanding of auxin-regulated plant growth and development.

**Figure 1:**
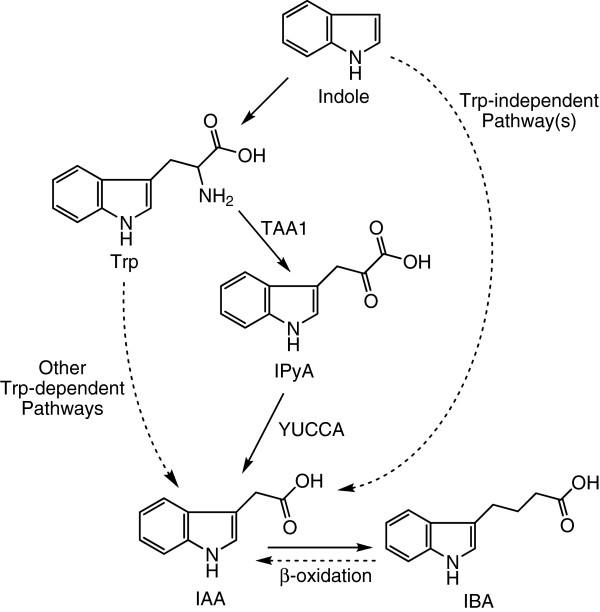
**A simplified summary of IAA biosynthetic pathways.** A solid line represents an enzymatic step. Dashed lines suggest multiple steps or pathways abbreviated. TAA1: Trp aminotransferase of Arabidopsis. YUCCA: an Arabidopsis flavin monooxygenase [3,11].

Among the different pathways of IAA regulation [3], the biosynthesis of IAA is a central way to regulate cellular IAA levels and has been actively studied for the past six decades [4]. In general, two types of pathways exist in plants to synthesize IAA: Trp-dependent and Trp-independent (Figure 1). The Trp-independent biosynthesis of IAA was suggested by a study showing low incorporation of ^15^N from ^15^N-indole]Trp into IAA [5] and was confirmed by studies showing the production of labeled IAA from its labeled precursors in Trp auxotrophic mutant plants [6,7]. Because no genes involved in Trp-independent pathways have been identified, analyzing the incorporation of labeled atoms into IAA and Trp from their common labeled precursors has been shown to be a useful tool to study the activity of Trp-independent IAA biosynthesis [6-10]. On the other hand, IAA can also be synthesized from multiple Trp-dependent pathways [11], and, importantly, several recent studies illustrated that in both Arabidopsis (*Arabidopsis thaliana*) and maize (*Zea mays* L.), IAA can be synthesized from Trp via the formation of indole-3-pyruvic acid (IPyA) [11-14] (Figure 1). Thus, IPyA has been demonstrated as an important intermediate of Trp-dependent IAA biosynthesis, and quantification of IPyA provides great potential to reveal mechanisms regulating this IAA biosynthetic pathway.

Indole-3-butyric acid (IBA) is another small molecule that displays auxin activity and has been identified as an endogenous plant compound [15]. Recent studies have confirmed that IAA can be produced from IBA via β-oxidation [16] and have shown that the IBA-derived IAA is important for plant development [17]. Additionally, the regulation of IBA metabolism also affects plant development and stress tolerance [18]. Therefore, analysis of IBA levels and its conversion to IAA also provides insights into the understanding of IAA regulation.

We previously reported a method for quantitative analyses of IAA and IBA, using solid phase extraction (SPE) columns and gas chromatography (GC) coupled with selected ion monitoring (SIM) on a single quadrupole mass spectrometer (MS) [19,20]. Here we describe an improved method that allows simpler equipment setup for sample preparation and smaller amounts of plant tissues collected for analysis, using selected reaction monitoring (SRM) on a GC triple-quadrupole MS (GC-MS/MS). In addition, this protocol describes methods for analyses of IAA precursors, including indole, Trp, and IPyA, and methods for rapid synthesis of stable-labeled internal standards. Thus, this protocol can be used to either measure levels of IAA and its precursors or to analyze IAA biosynthesis in plants.

## Materials

### Reagents

· Nitrogen gas [ultra high purity (UHP)]

· Helium gas (UHP)

· Argon gas (UHP)

· Hexane (HPLC grade; Fisher, cat. no. H302) ► *CAUTION*: flammable

· Ethyl acetate (HPLC grade; Fisher, cat. no. E195) ► *CAUTION*: flammable

· Acetonitrile (HPLC grade; Fisher, cat. no. A998) ►*CAUTION*: flammable

· Methanol (HPLC grade; Fisher, cat. no. A452) ►*CAUTION*: flammable

· Dichloromethane (Sigma-Aldrich, cat. no. 154792) ►*CAUTION*: harmful

· Pyridine (ACS grade; EMC Chemicals, cat. no. PX2020) ►*CAUTION*: flammable

· Diethyl ether (HPLC grade; Sigma-Aldrich, cat. no. 309966) ►*CAUTION*: flammable; forms peroxides

· [^13^C_6_]IAA (Cambridge Isotope Laboratories, cat. no. CLM-1896) ► *CRITICAL*: keep container tightly closed and store in a dry place at −20°C.

· [^13^C_8_,^15^N_1_]Indole (Cambridge Isotope Laboratories, cat. no. CNLM-4786)

· ^13^C_8_^15^N_1_IBA (synthesized from ^13^C_8_^15^N]indole as described in Additional file 1[19,21])

· [^**13**^C_**11**_,^**15**^N_**1**_]IPyA (synthesized from [^**13**^C_**11**_,^**15**^N_**2**_] Trp as described in Additional file 2)

· Sodium sulfate (Na_2_SO_4_, anhydrous; Sigma-Aldrich, cat. no. 239313)

· Sodium borodeuteride (NaB^2^H_4_; Sigma-Aldrich, cat. no. 205591)

· Methanol : water (8:1, v:v)

· Methanol : 8 M ammonium hydroxide (NH_4_OH; Fisher, cat. no. A669) (1:1, v:v)

· 0.3 N Sodium hydroxide (NaOH; Sigma-Aldrich, cat. no. S8045)

· 0.1 M Sodium bicarbonate, pH 7.0 (NaHCO_3_; Mallinckrodt, cat. no. 7412)

· 50 mM NaHCO_3_

· 0.2 M Imidazole, pH 7.0 (Sigma-Aldrich, cat. no. 56750) ►*CAUTION*: corrosive

NOTE: yellows with storage and yellowed material is not suitable for use. It may be recrystallized; otherwise should be discarded

· 50% (v/v) Isopropanol (HPLC grade; Fisher, cat. no. A451)

· Homogenization buffer: 65% isopropanol and 35% 0.2 M imidazole (pH 7.0)

· 0.25% Phosphoric acid (PA) (ACS grade; Fisher, cat. no. A242)

· 0.1 M Succinic acid, pH 6.0 (SA) (Sigma-Aldrich, cat. no. 224731)

► *CRITICAL*: store in refrigerator or prepare freshly, because bacteria grow well on succinic acid solutions.

· PA:SA (5:1, v:v), pH 3.0

► *CRITICAL*: store in refrigerator or prepare freshly, because bacteria grow well on succinic acid solutions.

· 25% (w/v) Polymethylmethacrylate epoxide resin (PMME, Macro-Prep; Bio-Rad, cat. no. 156–0000) suspension in 0.1 M NaHCO_3_ (pH 7.0)

· 25% (w/v) NH_2_ resin (Agilent, cat. no. 12213020) suspension in distilled water

· 25% (w/v) DOWEX® 50X2-400 ion-exchange resin (Sigma-Aldrich, cat. no. 217476), H^+^ form, suspension in distilled water

· 25% (w/v) Oasis® HLB resin (collected from HLB cartridges; Waters, cat. no. WAT106202) suspension in methanol

· Ethereal diazomethane (prepared as previously described [22,23])

· Methyl chloroformate (MCF; Sigma-Aldrich, cat. no. M35304) ►*CAUTION*: flammable, highly toxic by inhalation, toxic by ingestion, corrosive.

### Equipment

· MICROMAN positive-displacement pipettes (Gilson, cat. no. F148501, F148502, F148503, F148504, F148505, F148506)

· MICROMAN positive-displacement pipette tips (Gilson, cat. no. F148412, F148112, F148113, F148414, F148114, F148560)

· 10–200 μl Empty TopTip for solid phase extraction (SPE) and adaptors for centrifugation (Glygen, cat. no. TT2EMT)

· Repeater® plus positive displacement pipette (Eppendorf, cat. no. 022260201)

· Eppendorf Combitips plus (1.0 ml, 5.0 ml; Eppendorf, cat. no. 022266209, 022266403)

· Tungsten-carbide beads (2.38 mm; Craig Ball Sales, cat. no. CATU.002380.000.0010)

· SealRite® microcentrifuge tubes (0.5 ml, 2.0 ml; USA Scientific, cat. no. 1605–0000, 1620–2700)

· Teflon Mixer-Mill adapter for 1.5- to 2-ml microcentrifuge tubes (Qiagen, cat. no. 69984)

· Vibration Mill (Mixer-Mill; Qiagen, cat. no. MM300)

· Microcentrifuge (Eppendorf, cat. no. 5417R)

· 2-ml Screw capped micro tubes (Sarstedt, cat. no. 72.694)

· 2-ml Clear glass vials with polytetrafluoroethylene (PTFE)-lined caps (Fisher Scientific, cat. no. 03-391-7A)

· 2-ml Amber glass vials with PTFE-lined caps (Fisher Scientific, cat. no. 03-391-8A)

· 8-ml Clear glass vials with PTFE-lined caps (Fisher Scientific, cat. no. 03-391-7C)

· 4-ml Amber glass vials with PTFE-lined caps (Fisher Scientific, cat. no. 03-391-8B)

· Color pHast Strips (Fisher Scientific, cat. no. S60170)

· Wide-mouth crimp vials (Chrom Tech, cat. no. CTV-1104)

· 250 μl Glass inserts with bottom spring (Chrom Tech, cat. no. CTI-9425)

· Crimp cap with silicone rubber septum, PTFE coated (Chrom Tech, cat. no. 515011)

· Crimper for 11-mm crimp caps (Sigma-Aldrich, cat. no. 33195)

· Decapping pliers (Chrom Tech, cat. no. 904371)

· Micro-dissecting forceps (Sigma-Aldrich, cat. no. F4017)

· Gas chromatograph (GC)-mass spectrometer (MS): Trace GC Ultra with TriPlus autosampler, TSQ Quantum triple quadrupole MS (Thermo Scientific®)

· GC capillary column 1: HP-5 ms, 30 m, 0.25 mm diameter, 0.25 μm film thickness (Agilent Technologies, cat. no. 19091 S-433UI)

· GC capillary column 2 (for analysis of indole-3-pyruvic acid only): DB-17 ms, 30 m, 0.25 mm diameter, 0.25 μm film thickness (Agilent Technologies, cat. no. 122–4732)

### Equipment setup

#### Diazomethane derivatization equipment

The following items are needed:

· Glass diazomethane generator with clear-seal joints as described in Cohen [23]

· Teflon tubing (3 mm, Cole-Parmer, cat. no. C-06407-10)

· 8-ml Clear glass vials with PTFE-lined caps (Fisher Scientific, cat. no. 03-391-7C

· Stainless steel evaporator (six-position evaporator, Barvap 6, Zanntek, cat. no. 11–06000)

· Sand bath heated to 55°C

The stainless steel evaporator is connected with N_2_ gas tank by Teflon tubing, and the probes of the evaporator are positioned above the sand bath so that samples can be heated while being evaporated by flowing N_2_ gas. The Teflon tubing can be connected with the diazomethane generator during the diazomethane generation process [22,23].

#### SPE TopTips

**Figure 2:**
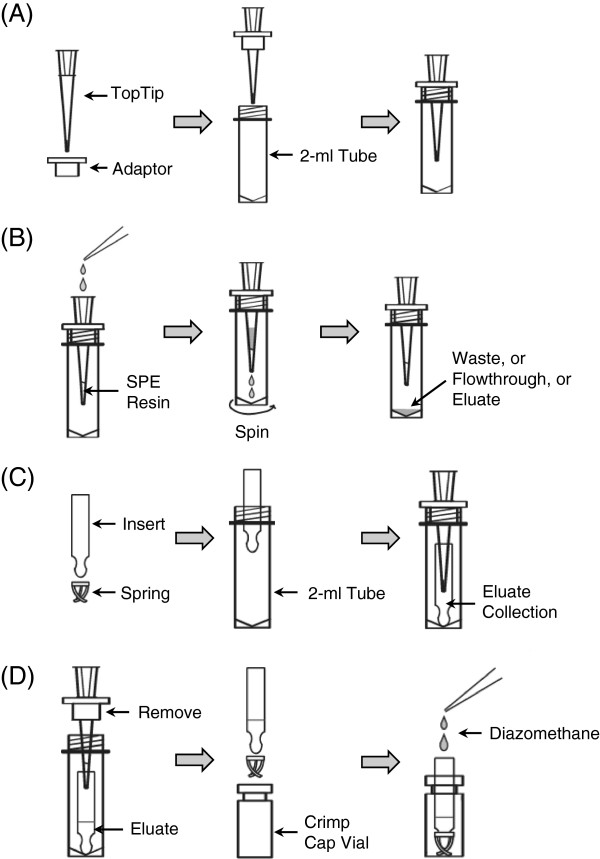
**Setup and utilization of TopTips for SPE extractions.** (**A**) Setup of a TopTip on a 2-ml centrifuge tube with an adaptor for centrifugation. (**B**) Process of liquid handling on a SPE TopTip. (**C**) Setup of a glass insert in a centrifuge tube for eluate collection. Corresponds to Step 45–46. (**D**) Process of methylating eluate in a glass insert. Corresponds to Steps 48–50.

Insert empty TopTips into adaptors that are placed on top of 2-ml screw capped micro tubes (Figure 2A). Load SPE resin suspension into each tip from the top of the tip. For NH_2_ resin, DOWEX® 50X2-400 ion-exchange resin, or Oasis® HLB resin, load 20 μl suspensions; for PMME resin, load 80 μl suspension. Then, spin the micro tubes together with the tips at 3,000 g for a few seconds (use “short” button on centrifuge). Repeat with higher centrifugal force and/or longer time if liquid does not pass though the tips.

#### GC-MS/MS system setup

The following items are used to set up the GC:

· Merlin Microseal™ high pressure kit (Thermo Scientific®, part no. 19050205, or contact Thermo Scientific® for the current part number)

· Merlin Microseal high pressure replacement septum (Merlin, part no. 410)

· Customized Teflon washer (made by Metro Industries, Inc. to the exact dimensions of the vespel/graphite washer supplied by Thermo Scientific®; a number of extras are available directly from our laboratory)

· 10 μl Syringe with an 80-mm needle (SGE Analytical Science, cat. no. 002989)

· Custom liners: Quartz glass tubes (outside diameter: 8 mm, inside diameter: 4 mm; part no. 4x8, Technical Glass Products, Inc.) cut to a length of 105 mm, lightly fire-polished and treated with Sylon CT™ (Sigma-Aldrich, cat. no. 33065-U) to deactivate the surface.

· Silanized quartz wool (Alltech, cat. no. 4233)

The GC was equipped with a split/splitless capillary inlet, and the standard septum was replaced by an adapter to accept the Merlin Microseal™ high pressure seal. To provide a better seal of the inlet and avoid adsorption of indolic compounds, the original vespel/graphite seal from the Thermo Scientific adapter kit was replaced by the custom Teflon washer, which was placed under the hexagonal adapter. A 10-μl syringe with an 80-mm needle was installed in the injector of the autosampler, and the injector position was adjusted to match the position and height of the Merlin Microseal™ valve. The original straight inlet liner (Restek, cat. no. 20939) was replaced by a custom quartz liner with a cluster of quartz wool inserted at the center. The MS was equipped with an electron impact (EI) source with an electron emission of 70 eV.

#### GC-MS/MS program

ISoftware Xcalibur^TM^ 2.1 (Thermo Scientific®, part. no. XCALI-64155) and TSQ Series 2.0.6 (Thermo Scientific®, part. no. XCALI-64251) were installed to operate the GC-MS/MS.

· TriPlus Autosampler: Sample volume was 1.0 μl; injection depth was standard; pre- and post-injection dwell time was 0 s; sampling depth mode was custom and sampling vial depth was 88%; sample type was “non viscous”.

· TRACE GC Ultra: in the “Oven” tab, under “Ramps”, the initial oven temperature was 70°C and the hold time was 2 minutes; the Ramp 1 rate was 20°C/min until temperature reached 280°C, and the hold time was 5 minutes. In the “Right SSL” tab, the inlet mode was splitless; the inlet temperature was 240°C; the split flow was 10 ml/min, and the splitless time was 1 min; the “constant septum purge” was checked. In the “Right Carrier” tab, the carrier gas was run under “constant flow” mode, and the flow rate was 1.0 ml/min; the “vacuum compensation” was checked. In the “Aux Zones” tab, the MS transfer line temperature was set to 280°C.

· TSQ Quantum (Condition 1): in the “EI/CI” tab, the number of states was 2; the state at start of run was “off”, and the state duration was 4 min; the emission current was 100 μA. The calibration gas setting was “off” and the CI method was unchecked. In the “scan editor” tab, the calibration correction method was unchecked; the MS acquire time was 17.50 min; scan type can be either SRM (selected reaction monitoring) or SIM (selected ion monitoring), and scan time was 0.025 s; the polarity was positive and the data type was centroid. When SRM mode was used, the argon collision gas pressure was 1.5 mTorr, and the collision energy was 10 V. The masses of ions to be monitored depended on the metabolites analyzed and will be described in the “procedure” section.

· TSQ Quantum (Condition 2, for analysis of indole-3-pyruvic acid only): similar to Condition 1, but the emission current in the “EI/CI” tab was 120 μA, the scan type in the “scan editor” tab was SRM, and the collision energy was 25 V.

## Protocol

### Plant sample preparation and homogenization

1. Collect plant material in 0.5-ml microcentrifuge tubes, and quickly freeze the tubes in liquid N_2_. If performing absolute quantification of endogenous metabolites, determine the exact fresh weight (FW) of plant material quickly and accurately before freezing the tubes. Keep tubes on dry ice or store them at −80°C.

· CRITICAL STEP: Sufficient plant material is necessary for the yield of satisfactory GC-MS/MS signals. Usually, 2–10 mg plant tissue is enough for the analysis of IAA/IBA, indole, and Trp; about 10 mg plant tissue is needed for the analysis of IPyA alone. Because IAA, IBA, indole, and Trp are extracted from the same aliquot of plant homogenate while IPyA is extracted from a separate aliquot of plant homogenate, this protocol assumes that ~20 mg plant tissue is collected as one sample.

· PAUSE POINT: Frozen samples can be stored at −80°C or shipped in a dry ice package.

2. Add 40 μl homogenization buffer and a tungsten carbide bead to each tube. If performing absolute quantification of endogenous compounds, add known amounts of internal standards together with homogenization buffer. Samples need to be kept on dry ice before next step.

· CRITICAL STEP: Add 20 μl homogenization buffer for a sample containing no more than 10 mg plant tissue. For every increment of 10 mg plant tissue, add an additional 20 μl homogenization buffer.

· CRITICAL STEP: For absolute quantification, accurate amounts of [^13^C_6_]IAA, [^13^C_8_,^15^N]IBA, [^13^C_8_,^15^N]indole, [^13^C_11_,^15^N]IPyA and (or) 100 ng of [^13^C_11_,^15^N_2_]Trp are added. To make sure that the detector’s response is within the linear range, the amount of internal standards should not exceed 10× of the endogenous level, or below 1/10 of the endogenous amount. For 20 mg of plant tissue, addition of 0.1-0.5 ng of each internal standard is suggested except that addition of 100–500 ng of [^13^C_11_,^15^N_2_]Trp is suggested.

3. Place the sample tubes into a vibration mill (with the Teflon adaptor and de-capped 2.0-ml microcentrifuge tubes placed in the adaptor), and homogenize for 4 min at 20–25 Hz. Repeat this step if tissues are not fully homogenized. If tissues are difficult to homogenize, add more tungsten carbide beads and increase the vibration frequency.

### Extraction and derivatization of IPyA

**Figure 3:**
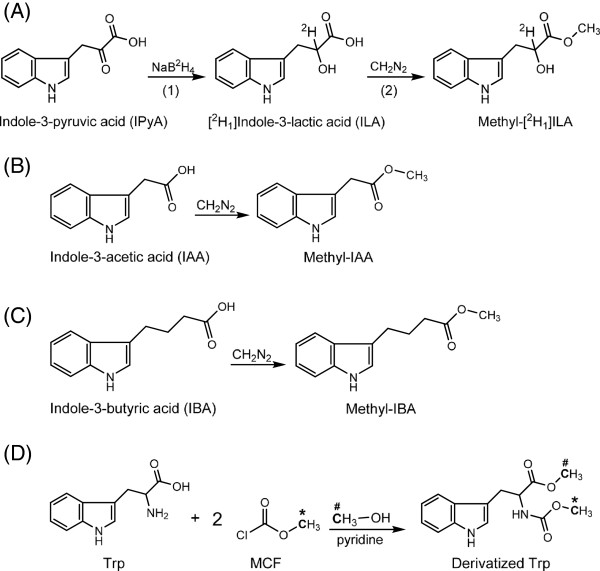
**Derivatizations of analytes prior to GC-MS analysis.** (**A**) IPA is first reduced by NaB^2^H_4_ to yield ^2^ H_1_ILA (Steps 5–6). ^2^ H_1_ILA is then methylated by diazomethane to form methyl-^2^ H_1_ILA (Step 20). (**B**) IAA and (**C**) IBA are methylated by diazomethane and yield methyl-IAA and methyl-IBA (Step 50). (**D**) Trp is derivatized by methyl chloroformate (MCF) in the presence of methanol and pyridine (Steps 60–61). Carbon atoms from both MCF and methanol are incorporated into the final product. The “*” and “#” labels mark the flow of these carbons [30].

4. Transfer 20 μl plant homogenate into a new tube. Leave the rest of plant homogenate on ice (see Step 24).

5. Add 8 μl 20 mg/ml NaB^2^H_4_ into each tube, and mix well (Figure 3A).

► *CRITICAL*: The 20 mg/ml NaB^2^H_4_ needs to be freshly prepared in 0.3 N NaOH, because NaB^2^H_4_ decomposes over time in aqueous solutions [24]. Perform this step soon after homogenization to avoid significant degradation of IPyA.

6. Incubate samples at 35°C for 1 h.

· PAUSE POINT: IPyA is reduced to [^2^H_1_]indole-3-lactic acid ([^2^H_1_]ILA), which is much more stable than IPyA, so samples can be stored at −20°C for a few days.

7. Add 5 μl 25% PA to acidify the sample and consume the residual NaB^2^H_4_.

8. Dilute the sample 10 fold by adding 200 μl distilled water and mixing well.

9. Check the pH of the diluted sample using a pH strip. The pH should be 2.5-3.0.

10. Wash the TopTips containing Oasis® HLB resin two times with 80 μl methanol and two times with 80 μl PA:SA (pH 3.0). Use repeater pipette to dispense the liquid into multiple TopTips. Liquid is forced to pass through TopTips by centrifugation at 3,000-6,000 g for a few seconds (Figure 2B).

► *CRITICAL*: The 2-ml screw capped micro tubes can hold up to 500 μl liquid before the liquid reaches the bottom of the TopTips. Therefore, discard the liquid waste when ~400 μl liquid has passed through TopTips. If 2-ml microcentrifuge tubes are used instead of screw capped tubes, less liquid can be held and the liquid waste needs to be discarded more frequently.

11. Spin the diluted samples at 12,000 g for 10 min.

12. Load the supernatant of each sample into individual TopTip.

13. Spin TopTips at 2,000 g to allow samples to pass through slowly and completely.

14. Wash the TopTips two times with 60 μl PA:SA (pH 3.0).

15. Wash the TopTips with 10 μl methanol. Spin the TopTips at 10,000 g for 1–2 min.

· CRITICAL STEP: The methanol wash and long period of centrifugation are designed to remove the water residue in the resin. If a significant amount of water is retained in the resin, the efficiency of methylation (by ethereal diazomethane) will be reduced, and the drying process will be extended.

16. Transfer the TopTips to new 2-ml tubes to collect the eluate.

17. Elute TopTips three times with 70 μl methanol.

18. Discard the TopTips.

19. Transfer the methanol eluate into a 2-ml clear glass vial.

· PAUSE POINT: Samples can be stored in capped vials at −20°C for a few days.

20. Fill each glass vial with ethereal diazomethane, and incubate for ~5 minutes (Figure 3A).

21. Evaporate the solvents to complete dryness using a gentle N_2_ gas stream in a sand bath heated to 55°C.

► *Caution*: Diazomethane is a toxic and explosive gaseous compound, and it is potentially explosive on contact with sharp edges such as scratched or broken glass. It should be prepared and used only in a fume hood and handled by hands protected with gloves. Read precautions in Cohen JD [23] and Barkawi LS and Cohen JD [22] before use.

22. Use 15 μl of ethyl acetate to re-suspend the sample, and transfer the ethyl acetate solution into a glass insert.

23. Place the glass insert into a crimp vial, and cap the vial.

· PAUSE POINT: Methylated samples can be stored at −20°C for a few days before GC-MS/MS analysis.

### Extraction of Indole, IAA, and IBA

24. Leave the rest of plant homogenate on ice for at least 1 h for isotopic equilibration.

· NOTE: This step follows Step 4

· PAUSE POINT: Homogenized samples can be stored at −20°C overnight.

25. Spin samples a short time to pellet plant homogenate debris to the bottom of the tubes.

26. Dilute the sample 10 fold by adding 180 μl distilled water and mix thoroughly.

27. Add 80 μl hexane and mix thoroughly.

28. Spin samples a short time to allow the organic and aqueous phases to separate clearly.

29. Transfer ~60 μl of the organic layer (upper layer) to a glass insert. Place the glass insert into a crimp vial, and cap the vial. This sample contains indole and can be analyzed by GC-MS/MS without derivatization.

· NOTE: Step 27–29 are designed to extract indole. These steps can be skipped if indole is not a targeted analyte. The samples of indole can be stored in sealed vials at 4°C in the dark for one week, but because indole is volatile do not let the sample dry.

30. Wash the TopTips containing NH_2_ resin sequentially with 60 μl of hexane, acetonitrile, ethyl acetate, 0.2 M imidazole, followed by three times with 100 μl of distilled water.

31. Transfer the TopTips to new 2-ml tubes to collect the flow-through from NH_2_ TopTips.

32. Spin the diluted samples at 12,000 g for 10 min.

33. Load the supernatant of each sample into individual TopTip.

34. Spin TopTips at 2,000 g to allow samples to pass through slowly and completely.

35. Transfer the TopTips to new 2-ml tubes. Cap the tubes containing the flow-through from the NH_2_ tips and store in a refrigerator. The flow-through contains Trp.

· NOTE: the flow-through is collected to extract Trp (See Step 53). It can be discarded if only IAA and IBA are of interest. The flow-through can be stored at 4°C for one month.

36. Wash the TopTips with 60 μl each of hexane and methanol.

37. Transfer the TopTips to new 2-ml tubes, each of which contains 25 μl of SA.

38. Elute IAA and IBA from the TopTips using 50 μl, 100 μl, and 50 μl PA, separately.

39. Discard the NH_2_ TopTips, and collect 2-ml tubes containing the acidic eluate.

40. Prewash the TopTips containing PMME resin two times with 100 μl methanol, followed by two times with 100 μl PA:SA.

41. Load the acidic eluate into the PMME TopTips.

42. Spin TopTips at 2,000 g to allow liquid to pass through slowly and completely.

43. Wash the TopTips three times with 60 μl PA:SA.

44. Wash the TopTips with 10 μl methanol. Spin the TopTips at 10,000 g for 1–2 min.

· CRITICAL STEP: Remove the water residue in the PMME resin to gain good methylation efficiency (similar to Step 15).

45. Remove the plastic spring from the bottom of the 250-μl glass inserts, and put the glass inserts into 2-ml screw capped micro tubes (Figure 2C).

46. Transfer the PMME TopTips to the micro tubes containing glass inserts. Make sure that the TopTips insert into the glass inserts (Figure 2C).

47. Elute IAA and IBA two times with 50 μl methanol. The eluate contains both IAA and IBA and is collected in the glass inserts in the micro tubes.

48. Discard the PMME TopTips.

49. Use the micro-dissecting forceps to take the glass inserts out of the micro tubes. Re-install the plastic spring at the bottom of the glass inserts, and put the glass inserts into crimp vials (Figure 2D).

· PAUSE POINT: Vials can be capped and stored at −20°C overnight.

### Derivatization of IAA and IBA

50. Add ethereal diazomethane into each glass insert until the insert is full, and incubate for ~5 minutes (Figure 2D; Figure 3B and 3C).

51. Evaporate the solvents until complete dryness using a gentle N_2_ gas stream in a sand bath heated to 55°C in a solvent hood.

► *Caution*: Diazomethane is toxic and potentially explosive (see Step 21).

52. Add 15 μl of ethyl acetate into each glass insert to re-suspend the methylated IAA and IBA. Cap the vials.

· PAUSE POINT: Methylated samples can be stored at −20°C for a few months or at −80°C for up to one year.

### Extraction and derivatization of Trp

53. Wash the TopTips containing DOWEX® 50X2-400 resins two times with 100 μl methanol and two times with 100 μl distilled water.

54. Load the TopTips with the flow-through from NH_2_ TopTips collected in Step 35.

55. Spin TopTips at 2,000 g to allow liquid to pass through slowly and completely.

56. Wash the TopTips with methanol : water (8:1, v:v).

57. Transfer the TopTips onto new 2-ml tubes.

58. Elute Trp two times with 100 μl methanol : 8 M NH_4_OH (1:1, v:v).

· PAUSE POINT: Trp eluate can be stored at 4°C for a few days.

59. Transfer 100 μl of the eluate containing Trp into a 2-ml clear glass vial.

60. In a fume hood, add 20 μl pyridine to each glass vial, and gently shake the vial to make sure the solvents mix.

61. Continuing in the fume hood, add 20 μl MCF into each vial. Wait for a minute to allow the reaction to complete (Figure 3D).

62. Sequentially add 300 μl each of 50 mM NaHCO_3_ and then dichloromethane into the vial. Cap the vial and mix thoroughly. Keep the vials still until two layers form clearly.

63. In a new 2-ml clear glass vial, add 10–20 mg anhydrous Na_2_SO_4_ (add enough to cover the bottom of the vial).

64. Transfer the dichloromethane layer (bottom layer) into the glass vial containing Na_2_SO_4_. Cap the vial. The dichloromethane contains derivatized Trp.

65. Wait for 30–60 min to allow Na_2_SO_4_ to absorb water from the dichloromethane solution.

· PAUSE POINT: Derivatized Trp is less stable than Trp, and it can be stored at −20°C for a few days. The actual time length for storage depends on the abundance of the compound in each sample. It is recommended to analyze the samples soon after derivatization.

66. Transfer 50 μl of the dichloromethane sample solution into a glass insert. Place the glass insert into a crimp vial, and cap the vial.

### GC-SRM-MS/MS analysis of derivatized IPyA

**Figure 4:**
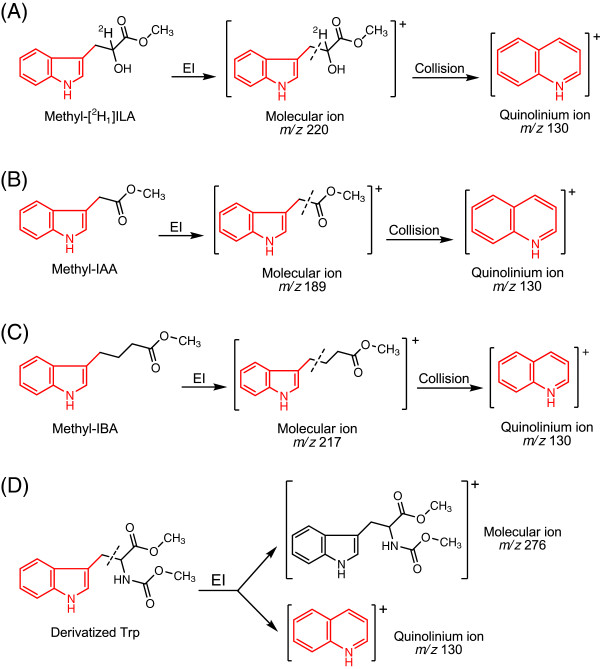
**Ionization and fragmentation of derivatized analytes during MS analyses.** EI: electron impact ionization source. (**A**) Methyl-[^2^ H_1_]ILA is first ionized and fragmented by EI. The molecular ion m/z 220 is selected and further fragmented to produce the quinolinium ion. Atoms in the molecular ion to the right of the dashed line are lost in the quinolinium ion. (**B**) Methyl-IAA and (**C**) methyl-IBA are first ionized by EI. The molecular ions m/z 189 and m/z 217 are selected and further fragmented to produce the quinolinium ions. Atoms in the molecular ions to the right of the dashed line are lost in the quinolinium ions. (**D**) Derivatized Trp is ionized and fragmented by EI. The molecular ion m/z 276 and quinolinium ion m/z 130 are the major products and are monitored by MS. Atoms in the original molecule to the right of the dashed line are lost in the quinolinium ion.

67. SRM is used as the acquisition mode, and the quinolinium ions produced from the molecular ions (Figure 4A) are monitored. For unlabeled methyl-[^2^ H_1_]ILA, the parent mass is 220 and the product mass is 130; for the methyl-[^13^C_11_,^15^N, ^2^ H_1_]ILA internal standard, the parent mass is 232 and the product mass is 140.

► *CRITICAL*: Because only the product (quinolinium) ions are monitored in the SRM mode, it is important to know what atoms are included in the quinolinium ions (Figure 4A) when stable-labeled IPyA is analyzed. Some stable-labeled atoms are missing from the quinolinium ions after fragmentation.

### GC-SIM-MS/MS analysis of indole

68. SIM is used as the acquisition mode and the molecular ions are monitored. The MS mode under “Scan Modes” is set to “Q1MS”. For the unlabeled indole, ion m/z 117 is monitored; for the [^13^C_8_,^15^N]indole, ion m/z 126 is monitored; for other types of labeled indole, the m/z of the monitored ion equals the nominal molecular mass.

### GC-SRM-MS/MS analysis of derivatized IAA and IBA

69. SRM is used as the acquisition mode and the quinolinium ions produced from the molecular ions (Figure 4B and 4C) are monitored. Under the “Run Settings” in the “Scan Editor” tab (of TSQ Quantum), the “Segments” are set to 2; “Segment Time” is 10.5 min for Segment 1 and 7.0 min for Segment 2. In Segment 1, methyl-IAA is monitored. For unlabeled methyl-IAA, the parent mass is 189 and the product mass is 130; for the methyl-[^13^C_6_]IAA internal standard, the parent mass is 195 and the product mass is 136. In Segment 2, methyl-IBA is monitored. For unlabeled methyl-IBA, the parent mass is 217 and the product mass is 130; for the methyl-[^13^C_8_,^15^N]IBA internal standard, the parent mass is 226 and the product mass is 139.

► *CRITICAL*: Because only the product (quinolinium) ions are monitored in the SRM mode, it is important to know what atoms are included in the quinolinium ions (Figure 4B and 4C) when stable-labeled IAA and IBA are analyzed. Some stable-labeled atoms are missing from the quinolinium ions after the fragmentation.

· CRITICAL STEP: methyl-IAA and methyl-IBA are analyzed in one GC-MS/MS run, and the GC retention time of Me-IBA is about 1 min longer than Me-IAA under the conditions described in the “Materials” section. Under these conditions, the retention time is about 10 min for Me-IAA and about 11 min for Me-IBA, so the division of the two segments in the run is set at 10.5 min. However, if different conditions are used (*e.g*., different GC column type or length, different oven temperatures), this setting needs to be adjusted based on the actual retention time of Me-IAA and Me-IBA.

### GC-SIM-MS/MS analysis of derivatized Trp

70. SIM is used as the acquisition mode, and both the molecular ions and the quinolinium product ions are monitored. For unlabeled Trp, the molecular ion m/z 276 and quinolinium ion m/z 130 (Figure 4D) are monitored; for the [^13^C_11_,^15^N_2_]Trp, the molecular ion m/z 289 and quinolinium ion m/z 140 are monitored. The quinolinium ions are much more abundant than the molecular ions, so the abundance of the quinolinium ions is used for quantification.

## Comments

### Development and principles of the protocol

**Figure 5:**
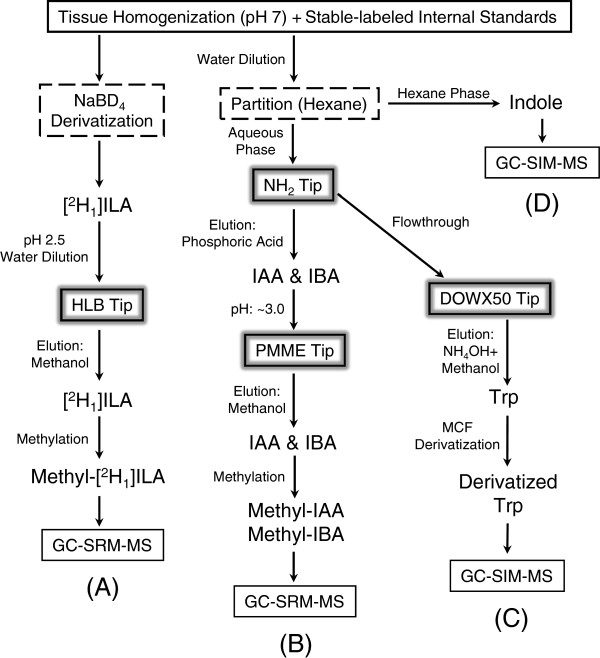
**Experimental procedures for extraction and derivatization of auxin and auxin precursors.** Tissue homogenization and addition of internal standards corresponds to Steps 2–3. Each homogenized sample is split into two aliquots. (**A**) Extraction and derivatization of indole-3-pyruvic acid (IPyA). Corresponds to Steps 4–23 and Step 67. (**B**) Extraction and derivatization of indole-3-acetic acid (IAA) and indole-3-butyric acid (IBA). Corresponds to Steps 24–26, 30–52, and Step 69. (**C**) Extraction and derivatization of tryptophan. Corresponds to Steps 53–66 and Step 70. **(D)** Extraction of indole. Corresponds to Steps 24–29 and Step 68.

The protocol for high-throughput SPE purification and GC-MS analysis has been validated as a good approach to quantify auxin levels and auxin biosynthesis [10,19,25]. Major procedures of the protocol are summarized in Figure 5. Briefly, indole, Trp, IAA, and IBA can be extracted from the same aliquot of plant homogenate and analyzed by GC-MS/MS separately, except that IAA and IBA are contained in the same sample and are analyzed in one GC-MS/MS run; using another aliquot of plant homogenate, IPyA can be extracted, derivatized, and analyzed by GC-MS/MS. The protocol does not need to be carried out completely as a set if only a particular analyte is of interest, and steps related to the individual analytes are outlined in Figure 5.

To quantify the level of endogenous compounds using isotope dilution, proper stable isotope labeled internal standards are required. An ideal internal standard should contain stable isotopes at non-exchangeable positions on compounds identical to the analytes, with mass increments of three or more [26]. When exchange of stable isotopes occurs during sample preparation and analysis, the amount of internal standard would decrease, leading to overestimation of the endogenous compound. When an internal standard with a small mass increment is used, the interference by natural abundance would greatly complicate the calculation (for example, ^13^C_1_indole-3-acetonitrile [27]). However, such stable-labeled internal standards are not always readily available, especially for IPyA which degrades rapidly in solution [25]. Therefore, we developed and describe herein protocols to rapidly synthesize ^13^C_8_^15^N_1_IBA from ^13^C_8_^15^N_1_indole (Additional file 1) and ^13^C_11_^15^N_1_IPyA from ^13^C_11_^15^N_2_Trp (Additional file 2). Considering the high cost of ^13^C_11_^15^N_2_Trp, [2,4,5,6,7-^2^ H_5_-indole]Trp can be a good alternative when GC-MS is used for the analysis [25]. However, when liquid chromatography-mass spectrometry (LC-MS) is used, the deuterium atoms on the indole ring may be lost during the ionization process [28]. Thus, deuterium-labeled indolic compounds are more complex to employ compared to ^13^C and/or ^15^N labeled compounds when used as internal standards with LC-MS as the stability of the deuterium-labeled internal standards needs to be carefully determined and verified. After proper stable-labeled internal standards are available, they should be introduced into plant samples at the earliest possible step, and as we described in the protocol, they are added together with the homogenization buffer right before tissue homogenization.

The SPE purification of IAA and IBA is derived from a protocol previously published [20], and the most significant improvement is the use of TopTips (Figure 2), which can retain SPE resin while letting liquid pass through the 1–2 μl slit at the bottom (http://www.glysci.com/products/TopTip.html). In addition to commercial TopTips, SPE tips can also be made by inserting small pieces of glass wool into regular pipette tips [29]. The first SPE tip used for IAA and IBA extraction contains amino (NH_2_) resin, which retains the ionized organic acids at neutral pH but not Trp, so Trp can be collected in the flow-through and extracted separately (Figure 5). The second SPE tip contains polymethylmethacrylate epoxide (PMME) resin, which binds protonated IAA and IBA when the pH is around 3.0 and releases them when methanol is added for elution. Finally, IAA and IBA are methylated by diazomethane in the presence of methanol (Figure 3), and the methylated IAA and IBA are analyzed by monitoring the quinolinium ion produced from the molecular ion (SRM; Figure 4) after GC separation. The sample recovery in the SPE steps varies from 80% to 90% [19]. It should be noted that although diazomethane is a hazardous compound, it has been proven to be very useful for chromatographic analysis (discussed previously [22]) with high yields and few byproducts. With proper handling, as described previously and herein, the generation, storage, and utilization of diazomethane have not led to any problem in our laboratory over the past 30 years.

The flow-through from the NH_2_ tip is collected to extract the IAA precursor Trp, using DOWEX50 resin [30]. If only amino acids are of interest, they can be extracted by loading diluted plant homogenate onto DOWEX50 tips, skipping the NH_2_ tip. DOWEX50 resin retains cations, such as the protonated amino group on Trp at neutral pH, and releases Trp when the pH is increased by ammonium hydroxide (NH_4_OH). Both the amino and carboxyl groups are then derivatized by methyl chloroformate (MCF) in the presence of methanol and pyridine at basic pH, based on a mechanism described previously [30] (Figure 3), and the derivatized Trp is analyzed by GC-MS using the SIM acquisition mode (Figure 4). The sample recovery in the SPE steps is around 90% [30].

The derivatization and extraction of IPyA is derived from methods previously described [25,31], with modifications that confer more rapid sample preparation. Because IPyA has a short half-life in solution, the ^13^C_11_^15^N_1_IPyA internal standard should be made just prior to use (Additional file 2). After tissue homogenization, IPyA is quickly derivatized by sodium borodeuteride (NaB^2^H_4_) to produce ^2^ H_1_indole-3-lactic acid (^2^ H_1_ILA, Figure 3), which degrades much more slowly and thus allows reliable quantification [25]. Because low levels of ILA exist in plants [32,33], it is necessary to use NaB^2^H_4_ (instead of NaBH_4_) to produce ^2^ H_1_ILA that can be distinguished from endogenous ILA by MS. It is important to understand that because about 11% of the plant endogenous ILA is ^13^C_1_ labeled from the natural occurrence of ^13^C, the existence of endogenous ILA can potentially still interfere with the analysis of IPyA. However, because the unlabeled quinolinium ion (m/z 130) from ^2^ H_1_ labeled IPyA (m/z 220) is monitored in the SRM method (Figure 4A), this reduces the potential to only 2-3% of the endogenous ILA that can interfere with the analysis of IPyA. Nevertheless, if high levels of endogenous ILA are present in the plant tissues of interest, these levels should be determined in a parallel extraction (discussed below) and their contribution to the measurement of IPyA should be subtracted. After converting IPyA to ^2^ H_1_ILA, the ^2^ H_1_ILA is protonated by acidifying the homogenate and extracted by Oasis® HLB resin, a hydrophilic-lipophilic-balanced reversed-phase sorbent (http://www.waters.com/waters/nav.htm?cid=513209). The carboxyl group of ^2^ H_1_ILA is also methylated by diazomethane (Figure 3), and the methylated product is analyzed by GC-MS using SRM (Figure 4). The sample recovery in the SPE steps is around 85% (defined as previously described [30]).

### Anticipated results

**Figure 6:**
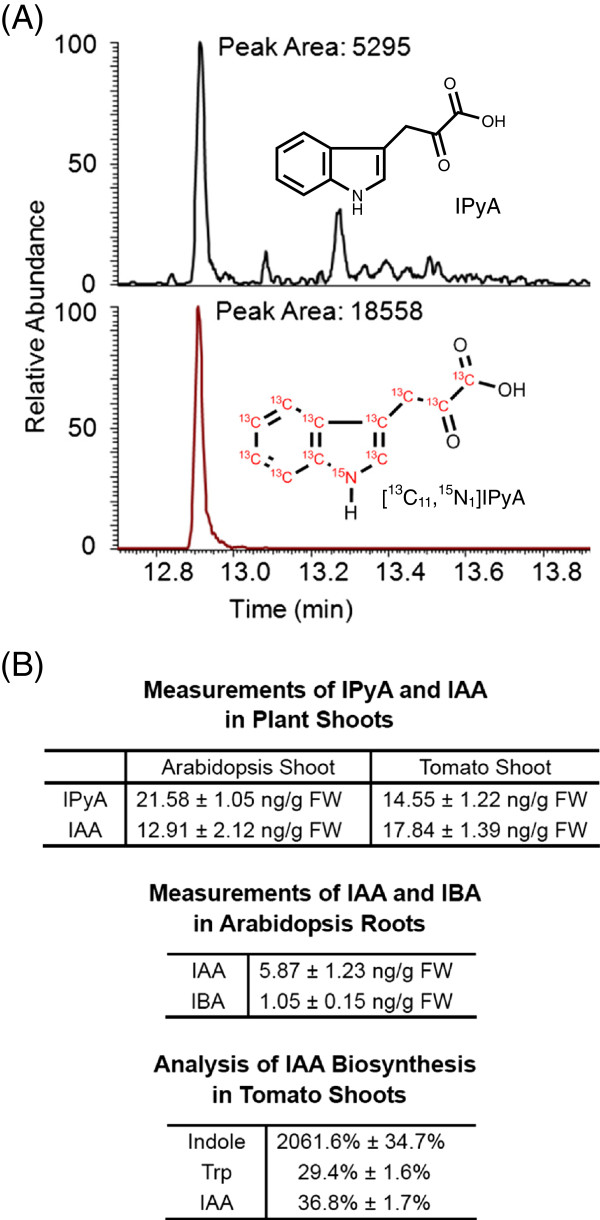
**Examples of GC-MS results from typical plant extracts.** (**A**) Chromatographic result from quantification of IPyA in 9.5 mg of Arabidopsis seedling tissue using GC-SRM-MS. (**B**) Measurements of plant endogenous IPyA, IAA, and IBA using different plant tissues, and analysis of IAA biosynthesis 4 hr after incubating tomato shoots with 0.1 mM [^13^C_1_]indole. One-week-old Arabidopsis seedlings and 5-day-old tomato seedlings were used for the studies. Average values of four replicates and standard errors are shown.

A group of results generated from extractions of IAA and IAA precursors is shown in Figure 6. The chromatographic results (Figure 6A) were displayed by Qual Browser in Xcalibur^TM^ 2.1 software, and the chromatogram of each ion (SIM) or reaction product ion (SRM) selected to be monitored is shown in each panel. Peaks were detected automatically by the software, and peak areas were also determined by the software. When necessary, peaks can also be determined manually by defining the start and end points of the peak.

All the chromatograms should be evaluated before calculations are applied. Each peak should be completely separated from its surrounding peaks, if any, and should be well resolved from the background, with a signal-to-noise ratio of greater than three. In general, it is more likely to have a second peak overlapping with the peak of analyte when the SIM acquisition mode is used, but this may also occur in the SRM acquisition mode. If peak overlapping forms a problem, samples can be analyzed again by GC-MS/MS using a different GC temperature gradient program (see Table 1).

**Table 1:** Common problems and suggested solutions

**Problems**	**Possible reasons**	**Solutions**
Liquid does not pass through TopTips before loading plant samples	The slit on TopTips is too narrow	Increase the centrifugal force to make liquid pass through. Or, switch to a new TopTip.
Liquid does not pass through TopTips after loading plant samples	Plant debris blocks the TopTip	Always try to avoid transferring plant debris into TopTips. Increase the centrifugal force to make liquid pass through. Use a dissecting probe to remove visible plant debris.
Drift of GC retention time	Deuterium labeled compounds are analyzed	It is normal for deuterium labeled compounds to have slightly shorter retention times.
	Carrier gas leaks significantly	Use a leak detector to find out where the leak is. Often, a worn Merlin Microseal septum is the source of the leak.
	Change of carrier gas	If the drift of GC retention time occurs after the change of carrier gas cylinders, check if correct gas cylinders are used.
Low yield of both endogenous IAA/IBA and IAA/IBA internal standard	Water in the methanol eluate reduces the methylation efficiency	If it takes a long time to evaporate the solvents to dryness, the samples are likely to contain residual water. Re-methylate samples by adding ethereal diazomethane and 10% methanol, and run samples again after drying and re-suspension.
	Wrong NH_2_ resin	Make sure that correct NH_2_ resin is used to extract IAA/IBA. Some brands of NH_2_ resin do not bind IAA/IBA sufficiently.
	The pH of solutions loaded onto PMME TopTips is too high	The pH has to be between 3 and 3.5. Cut a pH strip into narrower strips, and dip a strip in the solution to check the pH. Make sure that correct concentration of PA is made (pH ≤ 1.8), and avoid adding too much SA which increases the pH.
Low yield of endogenous IAA/IBA, but normal yield of IAA/IBA internal standard	Insufficient tissue homogenization and/or equilibration	Make sure that plant tissues are well homogenized. Allow longer time period for equilibration, e.g., overnight in the dark at 4 °C.
	Low endogenous IAA/IBA content in plant tissues	Collect more plant material for extraction. Usually, more plant material is required for IBA analysis as compared with IAA analyses.
Broad/tailed peaks	The GC liner is dirty	Change the liner, and cut ~30 cm from GC column from the injector end.
	The GC column is dirty	Turn off the MS, and change the GC column.
Overlapping peaks	The GC may need maintenance	See above.
	The plant sample may contain other metabolites that elute at similar GC retention time and produce ions with m/z values the same as the analytes	Run samples again using a different GC temperature gradient program. The temperature gradient commonly used is 20 °C/minute, and a slower or faster gradient can be used. Note that the retention time will be different when a different gradient is used. Run a standard to obtain the new retention time.
Reduced MS sensitivity	The tune file is out of date	Auto-tune the MS system.
	The EI source is dirty	Turn off the MS, and remove the parts of EI source. Reassemble the EI source after cleaning the parts.

For absolute quantification, the amount of plant endogenous compounds can be calculated from peak areas of the endogenous compound and the labeled internal standard. For example, in Figure 6A, IPyA from 9.5 mg plant tissue was extracted with 0.5 ng ^13^C_11_^15^N_1_IPyA. Because the amount of ^13^C_11_^15^N_1_IPyA was determined based on unlabeled IPyA using reverse isotope dilution (Additional file 2), the R-value discussed previously [20] is not necessary for the calculation here, and the sample shown contains 0.143 ng of IPyA, which is then divided by the fresh weight (FW) of the plant sample in order to calculate the concentration of IPyA. Therefore, the sample shown in Figure 6A contains 15.05 ng g^-1^ FW of IPyA. Results of IPyA measurements from Arabidopsis and tomato seedlings are also shown in Figure 6B, which are consistent with the previous reports [25,34]. Similarly, IAA and IBA from 2–10 mg plant tissues were extracted together with known amounts of ^13^C_6_IAA and ^13^C_8_^15^N_1_IBA. Using the isotope dilution equation that was fully discussed previously [20], the masses of endogenous IAA and IBA can be calculated (R-value: 1.13 for IAA; 1.33 for IBA, see Additional file 1), and they are then divided by the fresh weight of the plant sample to calculate concentrations of IAA and IBA. Results of IAA and IBA measurements from different plant tissues are shown in Figure 6B. In addition to Arabidopsis and tomatoes, using this method we have successfully analyzed IAA in different tissues from different plant species, including tobacco, corn, peas, *Lemna*, and *Angraecum sesquipedale*.

To study the activity of auxin biosynthesis, isotopic enrichment of stable-labeled auxin and auxin precursors can be analyzed. Figure 6B shows the enrichment of ^13^C in tomato shoots after incubation with 0.1 mM [^13^C_1_]indole for four hours. [^13^C_1_]indole is greatly enriched in the plant indole pool, suggesting that [^13^C_1_]indole was taken up into plants effectively. After subtraction of the natural abundance of [^13^C_1_]Trp (10%, a constant determined from unlabeled Trp), the average enrichment of [^13^C_1_]Trp synthesized from [^13^C_1_]indole was determined to be 29.4% (labeled Trp divided by unlabeled Trp). Similarly, after subtraction of the natural abundance of [^13^C_1_]IAA (10%, a constant determined from unlabeled IAA), the average enrichment of [^13^C_1_]IAA synthesized from [^13^C_1_]indole was determined to be 36.8%. Because the enrichment of [^13^C_1_]IAA synthesized from [^13^C_1_]indole exceeded the enrichment of [^13^C_1_]Trp synthesized from [^13^C_1_]indole, it can be concluded that at least a portion of [^13^C_1_]IAA was synthesized in the tomato plants via the Trp-independent biosynthetic pathway.

### Application of the method and experimental design

Firstly, this protocol can be used for absolute quantification of IAA and IAA precursors. Because the extraction and GC-MS analysis of IPyA is essentially extraction and analysis of its reduced product ILA, the method can also be used to quantify endogenous ILA using ^13^C_11_^15^N_1_ILA as the internal standard, which can be made by reducing synthesized ^13^C_11_^15^N_1_IPyA using NaBH_4_ (similar to Steps 17–22 in Additional file 2). Because isotope dilution is used in the quantification, the technical variation after the addition of internal standard is minimal, and thus the major variations are introduced before and during the sample homogenization step. The IAA content in plants can vary significantly depending on growth conditions and developmental stages [35-39], so plants should be grown under controlled environmental conditions and the tissues collected for analysis should be at similar developmental stages. Because the levels of IAA or its precursors are usually expressed as ng g^-1^ fresh weight of plant tissue, the tissue weight should be precisely determined before freezing, and water or soil attached to the plant surface should be removed before tissue collection. To minimize the effects of wounding, samples should be frozen in liquid N_2_ quickly after weighing and stored continuously at −80°C to avoid changes in IAA content until tissue homogenization. When homogenizing plant tissues, isopropanol is an important component in the homogenization buffer which denatures plant metabolic enzymes that can alter the content of IAA and other compounds in plant homogenates [40-42]. After homogenization, internal standards are added and plant metabolic enzymes are denatured by isopropanol, so the plant homogenate can then be manipulated or stored at a higher temperature with no alterations to the quantification results.

Additionally, the protocol can be used to quantify the enrichment of stable-labeled IAA synthesized from stable-labeled IAA precursors or putative precursors. For this type of analysis, plants should be incubated with stable-labeled compounds such as ^13^C_1_indole or ^13^C_8_^15^N_1_IBA for a certain period of time before tissue collection, and the abundance of both endogenous IAA and putative stable-labeled IAA should be monitored by GC-MS/MS. Because the enrichment is determined by the abundance of labeled IAA divided by abundance of endogenous IAA (see “anticipated result”), it is often not necessary to record the fresh weight of plant tissue or add internal standards prior to homogenization, unless the yield of labeled IAA is to be quantified. To quantify the amount of labeled IAA, the internal standard should contain a mass significantly different from the mass of labeled tracer IAA. For example, to quantify the amount of ^13^C_8_^15^N_1_IAA synthesized from ^13^C_8_^15^N_1_IBA, ^13^C_6_IAA was used as the internal standard [16]; and to quantify the amount of transported ^13^C_6_IAA in Arabidopsis hypocotyls, [4,5,6,7-^2^ H_4_-indole]IAA was added as the internal standard [43].

Using this protocol, the activity of IAA biosynthetic pathways can also be analyzed. By comparing the incorporation of ^13^C from ^13^C_1_indole into Trp and IAA pools (Figure 6, D and E), it is possible to identify biotic and abiotic factors that change the activity of Trp-independent and/or Trp-dependent IAA biosynthesis [9,10,38]. Similarly, by quantifying the enrichment of labeled IAA and IPyA, the significance of IPyA-dependent IAA biosynthesis under various biological conditions can be analyzed [44].

### Advantage of the method

A major advantage of this method is the improved detectability of analytes. The SRM acquisition mode confers an order of magnitude higher sensitivity compared with the SIM acquisition mode, so the amount of plant tissue required in this protocol is at least ten times less than in the previous method [20]. Based on analyses of chemical standards, the GC-SRM-MS/MS detection limit (signal-to-noise ≥ 3) is 0.01 pg/μl for IAA, 0.05 pg/μl for IBA, and 1 pg/μl for IPyA (weights of compounds were determined before derivatization). Considering the accuracy of weighing, a minimum amount of 2 mg tissue is recommended for quantification. This lower requirement for plant tissue allows faster tissue collection and tissue-specific analysis, which enables detection of localized changes in IAA content and IAA biosynthesis [10,45,46] and thus provides a better potential for understanding IAA-regulated biological processes. In addition, with the more sensitive assay, plants can be incubated with IAA precursors for shorter periods of time and still yield sufficient amounts of labeled IAA for detection while reducing artifacts generated by exogenous compounds [10,47]. Therefore, this more sensitive method can also provide more accurate assessments of IAA regulatory mechanisms.

To better quantify the trace amount of plant endogenous compounds in a high-throughput manner, we modified the inlet port of the GC (see “GC-MS/MS system setup”). We replaced the standard septum with a Merlin Microseal™ high pressure seal to reduce injection of the septum material into the GC column and permit less frequent change of the septum. We used a custom Teflon washer to provide a better seal between the Merlin seal adaptor and the GC inlet and to avoid interaction between the seal material and injected analytes. We also replaced the inlet liner with a custom quartz liner to minimize the effect of residual analytes from earlier injections carried-over to later injections and thus increased the accuracy of the assay.

Another highlight of the protocol is the use of SPE tips. Compared with SPE columns, which usually require a manifold connected with a vacuum pump for liquid manipulation, SPE tips can be manipulated by a standard microcentrifuge, allowing simpler instrumental setup, less solvent consumption/waste, and preparation of more samples at one time. Because the SPE resin can be packed into tips simply by adding the resin suspension, SPE tips can be easily customized, which significantly reduces the cost and greatly facilitates development of new methods. Based on our experimental design, we decided to use 200-μl TopTips, but other tip sizes such as 10-μl or 1000-μl tips can also be used when different sample sizes are to be applied.

## Conclusion

tlsb-.19pt?>The SPE tips and GC-MS/MS based method provides sensitive and accurate assays to quantify the plant hormone auxin, IAA, and its biosynthetic precursors including indole, Trp, IPyA, and IBA. The setup of SPE tips is simpler and more convenient compared with conventional SPE columns, and it also allows high-throughput extractions of trace amounts of small molecules. For an experienced researcher, roughly 48 samples can be prepared for analysis daily. The GC-MS/MS provides direct and precise quantification of plant endogenous IAA and its biosynthetic precursors using isotope dilution, and the amount of plant tissue required for the assay is small (typically 2–10 mg fresh weight). Additionally, the protocol can be used to analyze IAA biosynthesis and biosynthetic pathways using stable isotope labeling, and in the absence of complete knowledge of IAA biosynthetic mechanisms, stable isotope labeling enables comprehensive studies of IAA biosynthesis.

## Competing interests

The authors declare that they have no competing interests.

## Authors’ contributions

XL developed the protocol and wrote the paper. ADH and GG advised the research and edited the paper. JDC directed the research and edited the paper. All authors read and approved the final manuscript.

## Supplementary Material

Additional file 1 **Protocol: Preparation of [**^**13**^**C**_**8**_**,**^**15**^**N]indole-3-butyric acid (IBA).** Click here for file

Additional file 2 **Protocol: Preparation of [**^**13**^C_**11**_**,**^**15**^**N]Indole-3-pyruvic acid (IPyA).** Click here for file
