# Supplementary material for: Protocol: High-throughput and quantitative assays of auxin and auxin precursors from minute tissue samples
Source: Plant Methods. 2012 Aug 10;8:31. doi: 10.1186/1746-4811-8-31 (PMC3457856; doi:10.1186/1746-4811-8-31)
Supplement: Additional file 1 — Protocol: Preparation of [13C8,15N]indole-3-butyric acid (IBA). [file 1746-4811-8-31-S1.pdf]

Additional file 1-Protocol: Preparation of [$^{13}C_8$,^{15}N]indole-3-butyric acid (IBA)

Materials:

- [$^{13}C_8$,$^{15}N_1$]Indole
- Solid NaOH
- Ethyl acetate
- 50% Isopropanol
- γ-Butyrolactone (Sigma-Aldrich, cat. no. B103608) ►*CAUTION*: harmful
- Chloroform (HPLC grade; Sigma-Aldrich, cat. no. 650498) ►*CAUTION*: toxic
- 6 N Hydrogen chloride (HCl; Fisher Scientific, cat. no. A144-212) ►*CAUTION*: open in fume hood; protect eyes, hands, and clothing

Equipment:

- Teflon cup with cover, 23 ml (Parr Instruments, cat. no. A280-AC)
- Screw-top reaction bomb (Parr Instruments, cat. no. 276AC-T304-012304)
- Aluminum housed heating mantle (Glas-Col®, cat. no. 102B 5101977001)
- PowrTrol temperature control (Glas-Col®, cat. no. 104A PL912)
- Separatory funnels (250 ml, 500 ml; Fisher Scientific, cat. no. 10-436-1B, 10-436-1C)
- Rotary evaporator (Buchi, Rotavapor®, R-110)
- UV-visible spectrophotometer (Agilent 8453, cat. no. G1812AA)
- Quartz cuvette (LabShopOnline.com, cat. no. TCQ24)

Protocol:

1. In a Teflon cup, add 0.05 g of [$^{13}C_8$,$^{15}N_1$]indole, 3.2 g of NaOH, and 6.09 ml of γ-butyrolactone. It is common that the solid does not dissolve in the liquid.

2. Cover the Teflon insert and fit it into the screw-top reaction bomb. Close reaction bomb securely.

3. Heat the reaction bomb to 220 ºC at a rate of 2 ºC/minute in the heating mantle with the temperature control, and incubate at 220 ºC for a total time of 24 hours.

4. Turn off the temperature control and let the system cool to room temperature.

5. Dissolve the reaction mixture in the Teflon insert completely in ~50 ml distilled water.

6. Transfer the 50 ml reaction mixture solution into a 500-ml separatory funnel.

7. Add 120 ml chloroform in a separatory funnel to partition unreacted [$^{13}C_8$,$^{15}N_1$]indole.

8. Remove the chloroform phase (bottom phase).

9. Repeat Step 7-8 twice.

10. Transfer the aqueous layer to a beaker and adjust the pH to 2.5 by adding 6 N HCl.

11. Transfer the acidified solution into a 250-ml separatory funnel.

12. Add 50 ml ethyl acetate to partition the synthesis product.

13. Remove the aqueous layer (bottom layer) and collect the ethyl acetate layer.

14. Repeat Step 11-13 three times and combine the ethyl acetate extracts.

15. Evaporate the ethyl acetate using a rotary evaporator.

16. Re-dissolve the product in a minimal amout of 50% isopropanol. This concentrated [$^{13}C_8$,$^{15}N_1$]IBA solution can be stored at -20 ºC for a few years.

17. Verify the product by GC-MS using "Full Scan" mode after diazomethane methylation (see the procedure in the main text). An example spectral scan is shown in **Additional Figure 1A**, which illustrates the identity and purity of the labeled compound. The product can be further purified by reverse phase liquid chromatography, and the R-value can be calculated using the equation shown in **Additional Figure 1B**. Similar to IAA [20], the concentration of IBA can be determined by spectrophotometry, with λ_{max} at 282 nm and an absorption coefficient of 6,060 M^{-1} cm^{-1}.

(A)

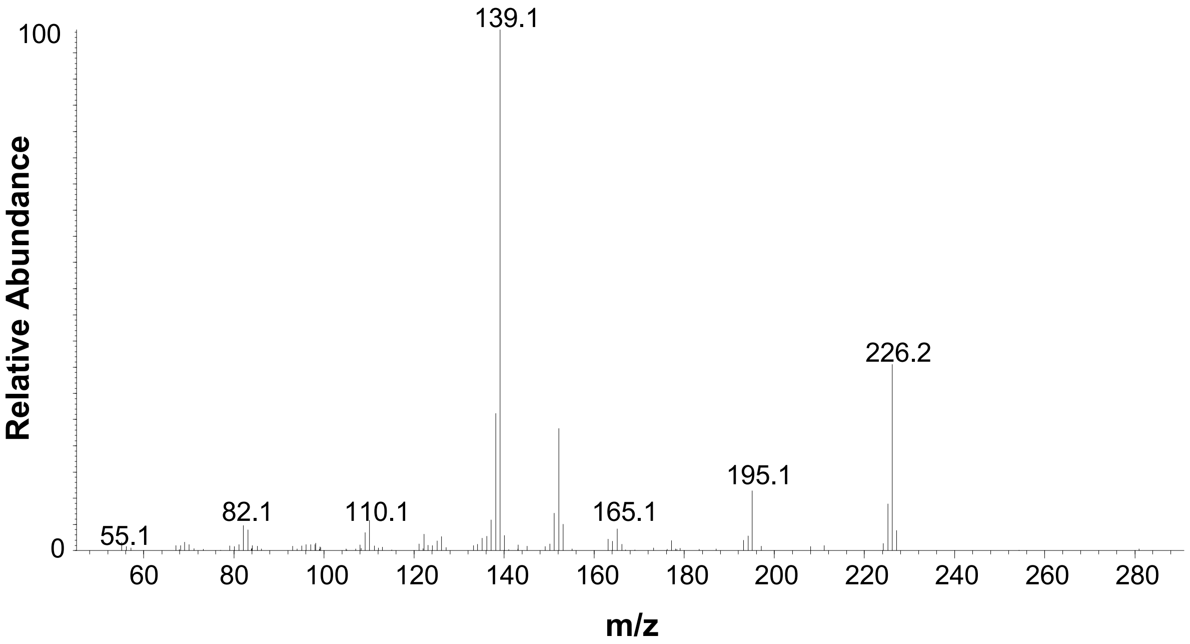

(B)

$$R = \frac{\left(\dfrac{m/z\ 130}{\sum m/z\ 130,131,132} \right)_{unlabeled}}{\left(\dfrac{m/z\ 139}{\sum m/z\ 130,131,132,133,134,135,136,137,138,139} \right)_{labeled}}$$

Additional Figure 1 Analyses of [$^{13}C_8$,^{15}N]IBA internal standards. **(A)** A full-scan mass spectrum showing ions generated from Me-[$^{13}C_8$,$^{15}N_1$]IBA. **(B)** Equation showing the calculation of the R-value.
