# Supplementary material for: Protocol: High-throughput and quantitative assays of auxin and auxin precursors from minute tissue samples
Source: Plant Methods. 2012 Aug 10;8:31. doi: 10.1186/1746-4811-8-31 (PMC3457856; doi:10.1186/1746-4811-8-31)
Supplement: Additional file 2 — Protocol: Preparation of [13C11,15N]Indole-3-pyruvic acid (IPyA). [file 1746-4811-8-31-S2.pdf]

Additional file 2-Protocol: Preparation of [$^{13}C_{11}$,^{15}N]Indole-3-pyruvic acid (IPyA)

► *CRITICAL*: For absolute quantification of IPyA in biological samples, a known amount of [$^{13}C_{11}$,^{15}N]IPyA is added to each sample as the internal standard, and [$^{13}C_{11}$,$^{15}N_1$]IPyA has to be synthesized just prior to or no more than 2 days before use.

Materials:

- 50 mM Phosphate buffer (pH 8.0), made from sodium phosphate monobasic (NaH_2PO_4, Sigma-Aldrich, cat. no. S8282) and sodium phosphate dibasic (Na_2HPO_4; Sigma-Aldrich, cat. no. S7907)
- 0.2 mg/ml Transaminase (Sigma-Aldrich, cat. no. T7684) in 50 mM phosphate buffer
  ► *CRITICAL*: store as 100 µl aliquots at -20 ºC. Use one aliquot for each reaction.
- [$^{13}C_{11}$,$^{15}N_2$]L- Trp (Cambridge Isotope Laboratories, cat. no. CNLM-2475)
- α-Ketoglutarate (Sigma-Aldrich, cat. no. K-1750)
- 5 mM Pyridoxal 5′-phosphate (PLP; Sigma-Aldrich, cat. no. 82870)
- 10 mM Ascorbic acid (Sigma-Aldrich, cat. no. A4544)
- 25% Phosphoric acid (PA) (ACS grade; Fisher, cat. no. A242)
- Indole-3-pyruvic acid (Sigma-Aldrich, cat. no. I7017)
  ► *CRITICAL*: the compound is sensitive to oxygen. Keep container tightly closed and store in a dry place at -20 ºC.
- Ethyl acetate
- 50% Isopropanol

Synthesis of [$^{13}C_{11}$,^{15}N]IPyA using an enzyme-catalyzed transamination reaction

1. Let one aliquot of transaminase solution thaw on ice.

2. In 1 ml of 50 mM phosphate buffer (pH 8.0), add 1 mg [$^{13}C_{11}$,$^{15}N_2$]Trp and 1 mg α-ketoglutarate.

3. Add 20 µl 5 mM PLP and one aliquot of transaminase solution.

4. Mix the solution gently by inverting the tube several times.

5. Incubate the reaction mixture at 37 ºC in the dark for 3 h.

6. Add 1 ml 10 mM ascorbic acid and 50 µl 25% PA to bring the pH to 2.5.

7. Add 600 µl ethyl acetate to partition the synthesized [$^{13}C_{11}$,^{15}N]IPyA.

8. Collect ethyl acetate (upper layer) into a 2-ml amber glass vial.

9. Add another 600 µl ethyl acetate to partition, and collect the ethyl acetate into the same amber glass vial.

10. Evaporate ethyl acetate to complete dryness using a gentle N_2 gas stream in a sand bath heated to 55 ºC.

11. Re-suspend the [$^{13}C_{11}$,^{15}N]IPyA in 1 ml 50% isopropanol. The product can be verified by GC-MS using a "Full Scan" mode after $NaBH_4$ derivatization (see the procedure in the main text). An example spectral scan is shown in **Additional Figure 2**, which illustrates the identity and purity of the labeled compound.

12. Cap the vial and store the solution at -20 ºC.

13. The yield of [$^{13}C_{11}$,^{15}N]IPyA is about 0.1 mg.

► *CRITICAL*: The concentration of the [$^{13}C_{11}$,^{15}N]IPyA solution has to be determined immediately before use (Step 2 in "Procedure").

Determine the concentration of [$^{13}C_{11}$,^{15}N]IPyA solution by reverse isotope dilution

14. Freshly prepare 0.1 mg/ml unlabeled IPyA in 50% isopropanol.

15. Freshly prepare 20 mg/ml NaB^2H_4 in 0.3 N NaOH.

16. In a 0.5-ml microcentrifuge tube, accurately add 5 µl each of unlabeled IPyA and synthesized [$^{13}C_{11}$,^{15}N]IPyA solution.

➢ CRITICAL STEP: for the ease of calculation in Step 15, it is recommended to add equal volumes of IPyA and [$^{13}C_{11}$,^{15}N]IPyA solutions.

17. Add 8 µl NaB^2H_4 solution and mix well.

18. Incubate the tube at 37 ºC for 30 min.

19. Add 5 µl 25% PA to acidify the mixture and consume the residual NaB^2H_4.

20. Add 200 µl water and mix well.

21. Extract the reduced IPyA (now ILA) by adding 100 μl ethyl acetate to partition.

22. Collect the ethyl acetate extract in a 2-ml clear glass vial.

23. Add 100 μl methanol to the vial.

24. Methylate the sample by filling the vial with ethereal diazomethane.

25. Evaporate the solvents until complete dryness using a N_2 gas stream.

26. Re-suspend the compounds in 1 ml of ethyl acetate.

27. Analyze the sample using GC-MS/MS under SRM mode. For unlabeled methyl-ILA, the transition of parent ion m/z 220 to the product ion m/z 130 is monitored; for methyl-[$^{13}C_{11}$,^{15}N]ILA, the transition of parent ion m/z 232 to the product ion m/z 140 is monitored.

28. The ion abundance ratio of m/z 130 over m/z 140 equals the ratio of IPyA concentration over [$^{13}C_{11}$,^{15}N]IPyA concentration in the initial sample.

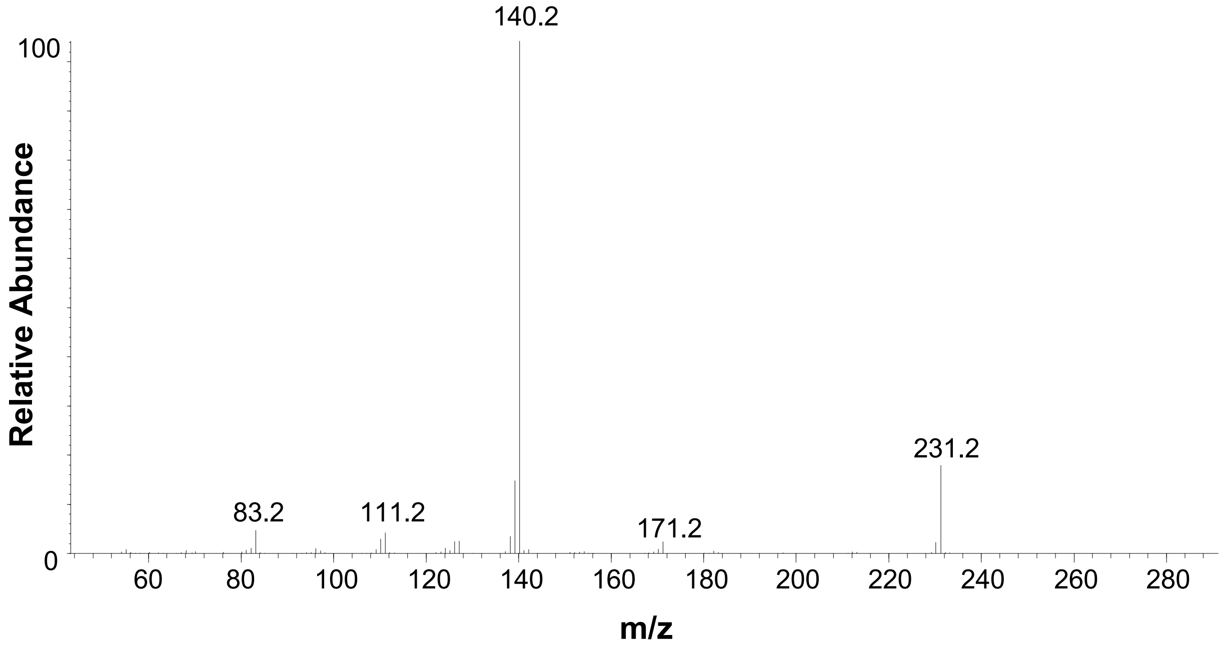

Additional Figure 2 Analyses of [$^{13}C_{11}$,^{15}N]IPyA internal standards. A full-scan mass spectrum showing ions generated from Me-[$^{13}C_{11}$,^{15}N]ILA.
